# Chronic Inhibition of 11**β**-Hydroxysteroid Dehydrogenase Type 1 Activity Decreases Hypertension, Insulin Resistance, and Hypertriglyceridemia in Metabolic Syndrome

**DOI:** 10.1155/2013/427640

**Published:** 2013-03-18

**Authors:** Christine G. Schnackenberg, Melissa H. Costell, Daniel J. Krosky, Jianqi Cui, Charlene W. Wu, Victor S. Hong, Mark R. Harpel, Robert N. Willette, Tian-Li Yue

**Affiliations:** Heart Failure Discovery Performance Unit, Metabolic Pathways and Cardiovascular Therapeutic Area, GlaxoSmithKline, UW2521, P.O. Box 1539, 709 Swedeland Road, King of Prussia, PA 19406-0939, USA

## Abstract

Metabolic syndrome is a constellation of risk factors including hypertension, dyslipidemia, insulin resistance, and obesity that promote the development of cardiovascular disease. Metabolic syndrome has been associated with changes in the secretion or metabolism of glucocorticoids, which have important functions in adipose, liver, kidney, and vasculature. Tissue concentrations of the active glucocorticoid cortisol are controlled by the conversion of cortisone to cortisol by 11**β**-hydroxysteroid dehydrogenase type 1 (11**β**-HSD1). Because of the various cardiovascular and metabolic activities of glucocorticoids, we tested the hypothesis that 11**β**-HSD1 is a common mechanism in the hypertension, dyslipidemia, and insulin resistance in metabolic syndrome. In obese and lean SHR/NDmcr-cp (SHR-cp), cardiovascular, metabolic, and renal functions were measured before and during four weeks of administration of vehicle or compound 11 (10 mg/kg/d), a selective inhibitor of 11**β**-HSD1. Compound 11 significantly decreased 11**β**-HSD1 activity in adipose tissue and liver of SHR-cp. In obese SHR-cp, compound 11 significantly decreased mean arterial pressure, glucose intolerance, insulin resistance, hypertriglyceridemia, and plasma renin activity with no effect on heart rate, body weight gain, or microalbuminuria. These results suggest that 11**β**-HSD1 activity in liver and adipose tissue is a common mediator of hypertension, hypertriglyceridemia, glucose intolerance, and insulin resistance in metabolic syndrome.

## 1. Introduction

Metabolic syndrome is a constellation of interrelated risk factors that promote the development of cardiovascular disease. The National Cholesterol Education Program (NCEP) Expert Panel on Detection, Evaluation, and Treatment of High Blood Cholesterol in Adults (Adult Treatment Panel III) defined the characteristics of the metabolic syndrome as elevated blood pressure, insulin resistance (with or without glucose intolerance), abdominal obesity, atherogenic dyslipidemia (elevated triglycerides, small LDL particles, and low HDL cholesterol), and prothrombotic and proinflammatory states [1]. Rather than any single factor, the Adult Treatment Panel III specified that combination of three out of five of these factors must be present to establish a diagnosis of metabolic syndrome. These factors include elevated blood pressure, elevated fasting glucose, elevated triglycerides, reduced HDL cholesterol, and abdominal obesity [1, 2]. Using these criteria in population-based studies, investigators reported that the prevalence of metabolic syndrome is increasing and contributes to higher rates of cardiovascular events [3, 4]. However, it remains unclear whether these interrelated risk factors share a common regulatory mechanism.

Glucocorticoids such as cortisol are important mediators in the regulation of cardiovascular and metabolic functions. Through activation of glucocorticoid or mineralocorticoid receptors, glucocorticoids impact vascular, adipose, liver, and kidney functions [5, 6]. Some of the glucocorticoid activities include gluconeogenesis, liposynthesis, insulin resistance, accumulation of visceral fat, vascular reactivity, vascular remodeling, and sodium reabsorption [5–9]. Prospective, cross-sectional studies on humans have shown that plasma cortisol or 24-hour renal cortisol excretion is correlated with some of the risk factors of metabolic syndrome [10–12]. The pathophysiological importance of glucocorticoid activity in metabolic disorders is exemplified in patients with Cushing's syndrome who have abnormally high plasma cortisol/cortisone ratio, which results from either administration of glucocorticoids or increased adrenal secretion of cortisol, and develop hypertension, obesity, and insulin resistance [13, 14]. Importantly, the ability to therapeutically reverse hypertension and other features of Cushing's syndrome with the antiglucocorticoid agent RU486 [6] suggested that limiting the actions of cortisol may be an important mechanism for controlling the development and maintenance of hypertension and other cardiovascular risk factors in metabolic syndrome. The regulation of cortisol activity is controlled by the local action of the microsomal enzyme 11*β*-hydroxysteroid dehydrogenase within tissues. There are two isozymes of 11*β*-hydroxysteroid dehydrogenase: type 1 (11*β*-HSD1) converts inactive cortisone to active cortisol and type 2 (11*β*-HSD2) converts active cortisol to inactive cortisone. 11*β*-HSD1 is most abundantly expressed in liver and adipose tissue [15, 16]. In contrast, 11*β*-HSD2 is mainly expressed in mineralocorticoid target tissues such as the kidney, colon, salivary, and sweat glands [17] where the enzyme prevents activation of the mineralocorticoid receptor by cortisol. 

 Inhibition of 11*β*-HSD2 activity causes hypertension and hypokalemia [18, 19]. However, the contribution of 11*β*-HSD1 to blood pressure regulation, especially in the context of its role in metabolic syndrome, is less clear. Genetic expression levels of 11*β*-HSD1 have been associated with blood pressure regulation in preclinical studies [20–22]. For example, mice with genetic overexpression of 11*β*-HSD1 have high blood pressure but mice with genetic knockout of 11*β*-HSD1 are normotensive. Clinical studies of an 11*β*-HSD1 inhibitor have shown mixed blood pressure results [23, 24].

To directly test the hypothesis that 11*β*-HSD1 is a common mechanism in the hypertension, dyslipidemia, and insulin resistance found in metabolic syndrome, we compared the cardiovascular, renal, and metabolic effects of a pharmacological inhibitor of 11*β*-HSD1 within the context of a preclinical setting of metabolic syndrome. The leptin receptor deficient spontaneously hypertensive rat (SHR-cp) is a well-established model of metabolic syndrome with hypertension, dyslipidemia, insulin resistance, and obesity [25–27]. Our findings of improved global function by the 11*β*-HSD1 inhibitor compound 11 [28] in this model support a role of 11*β*-HSD1 as a coordinated regulator of these diverse processes of metabolic syndrome.

## 2. Methods

### 2.1. Animals

Animal procedures were approved by the Institutional Animal Care and Use Committee of GlaxoSmithKline and were in accordance with NIH Guidelines for the Care and Use of Animals. Adult male littermates of obese (cp/cp) and lean (+/+) SHR/NDmcr-cp (SHR-cp, Vassar College) rats aged 4-5 months were used in all studies. Rats were anesthetized, surgically implanted with radiotelemetry catheters (DSI) in the abdominal aorta, and allowed to recover for at least one week before baseline measurements were taken. After cardiovascular, renal, and metabolic functions were determined at baseline, obese and lean SHR-cp were divided into two groups each. Groups were administered vehicle (1% DMSO, 6% Cavitron; *n* = 12 obese, *n* = 9 lean) or compound 11 at 10 mg/kg/d (*n* = 13 obese, *n* = 10 lean) via gavage for 4 weeks. The doses of compound 11 were chosen based on previously published studies [28]. Liver, visceral adipose tissue, and kidney were harvested at the end of the study, rapidly frozen in liquid nitrogen, and stored at −80°C.

Mean arterial pressure and heart rate were measured directly in conscious rats using radiotelemetry before and during vehicle or compound 11 administration. Blood pressure and heart rate were collected every 5 minutes for 22 hours daily and averaged. Urine was collected over 24 hours from rats individually housed in metabolic cages and stored at −80°C until analysis. Plasma was collected at the end of the urine collection for determination of plasma electrolyte, hormone, and creatinine concentrations. Plasma lipids, insulin, and blood glucose concentrations were determined in overnight-fasted rats. Plasma insulin was measured using ELISA (LINCOplex). Whole blood glucose was measured immediately upon sampling using a glucometer (Accu-Chek Advantage). Plasma aldosterone concentration was measured by ^125^I-radioimmunoassay (Siemens). Plasma renin activity was measured by ^125^I-radioimmunoassay (Diasorin). Electrolytes, creatinine, microalbumin, cholesterol, triglycerides, high-density lipoprotein (HDL), low-density lipoprotein (LDL) and nonesterified fatty acids (NEFA) were measured using an Olympus AU640 Clinical Analyzer. 

### 2.2. Oral Glucose Tolerance Test

Oral glucose tolerance testing was conducted before and 4 weeks after vehicle or compound 11 administration in lean and obese SHR-cp. Rats were fasted overnight before challenge with an oral glucose load as previously described [29]. Briefly, blood samples were collected from conscious rats at baseline and 15, 60, and 120 minutes after oral administration of 2 g D-glucose/kg body weight. 

### 2.3. Preparation of Tissue Microsomes

Microsomes were prepared from harvested liver, visceral adipose tissue, and kidney according to the method reported previously [30]. Briefly, the harvested tissues were homogenized using a polytron in a buffer containing 50 mM Tris HCl, 150 mM KCl, and 2 mM EDTA (pH 7.4). The volume of homogenizing buffer was determined by the weight of thawed tissue (1 gram of tissue: 4 mL of buffer). The homogenate was centrifuged at 13,000 g for 20 minutes, and the supernatant was further centrifuged at 109,000 g for 60 minutes at 4°C. The supernatant was discarded and the pellet was resuspended in 250 mM sucrose (0.25 mL/g tissue) and stored at −80°C prior to use.

### 2.4. Measurements of 11*β*-Hydroxysteroid Dehydrogenase Type 1 and Type 2 Activities

The measurements of 11*β*-HSD1 activity in liver and adipose tissue and 11*β*-HSD2 activity in kidney were performed using a scintillation proximity assay (SPA) as reported previously [31, 32]. Briefly, for the 11*β*-HSD1 assay, 40 *μ*L of [^3^H]-cortisone diluted in 80 nM in assay buffer (50 mM HEPES, 100 mM KCl, 5 mM NaCl, 2 mM MgCl_2_, pH 7.4) with 1 mM NADPH was dispensed to a 96-well plate. To start the reaction, 10 *μ*L of tissue microsome preparation (adipose 200 *μ*g/mL; liver 10 *μ*g/mL) was added to each well. As a control to determine assay background, assay buffer was added instead of microsomes. The plate was shaken briefly and incubated at 37°C for 2 hours. Meanwhile, a stop solution containing 5 mg/mL protein A-coated YS SPA beads resuspended in Superblock (Pierce), 10 *μ*M 18*β*-glycyrrhetinic acid, and 1 *μ*g/mL monoclonal cortisol antibody (East Coast Biologics) was prepared and incubated for 2 hours at room temperature to form the SPA bead complex. For each well, 70 *μ*L of the stop solution containing SPA beads was added to terminate the enzyme reaction. The plate was then incubated for another 2 hours. The signal emitted by the SPA/product complex was measured on a TopCount (Packard). 

For the 11*β*-HSD2 assay, 40 *μ*L of [^3^H]-cortisol diluted in 80 nM in assay buffer with 2 mM NAD was dispensed to a 96-well plate. 10 *μ*L of kidney microsomes (30 *μ*g/mL) was added to the plate. The plate was shaken briefly and incubated at 37°C for 1.5 hour. The stop solution was prepared as described above, and 70 *μ*L of the stop solution containing SPA beads was dispensed to all wells to terminate the enzyme reaction. The plate was then incubated for 1 hour at room temperature while slightly shaking to allow the capture of the remaining substrate, [^3^H]-cortisol, by the SPA bead complex. The signal emitted by the SPA/product complex was measured on a TopCount (Packard).

### 2.5. Statistical Analysis

Data are reported as mean ± SEM. Analysis of variance followed by Bonferroni multiple comparison test or Student's *t*-test was used to evaluate statistical significance. *P* < 0.05 was considered to be statistically significant.

## 3. Results

### 3.1. Cardiovascular and Renal Function

**Figure 1:**
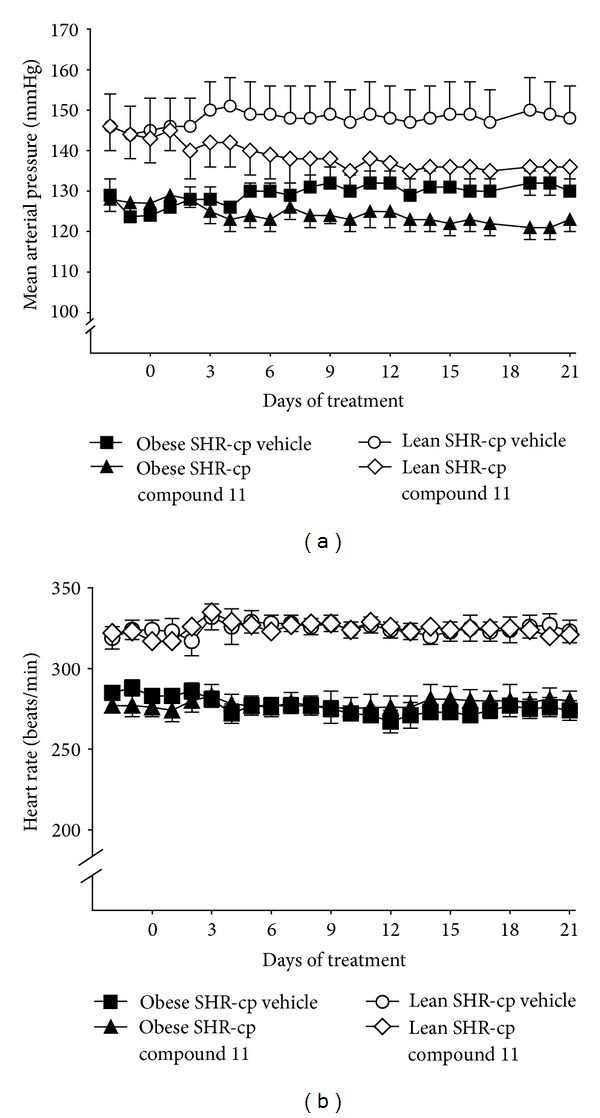
Absolute mean arterial pressure and heart rate in conscious, chronically instrumented obese and lean SHR-cp before and during chronic administration of vehicle or compound 11 (10 mg/kg/d).

**Figure 2:**
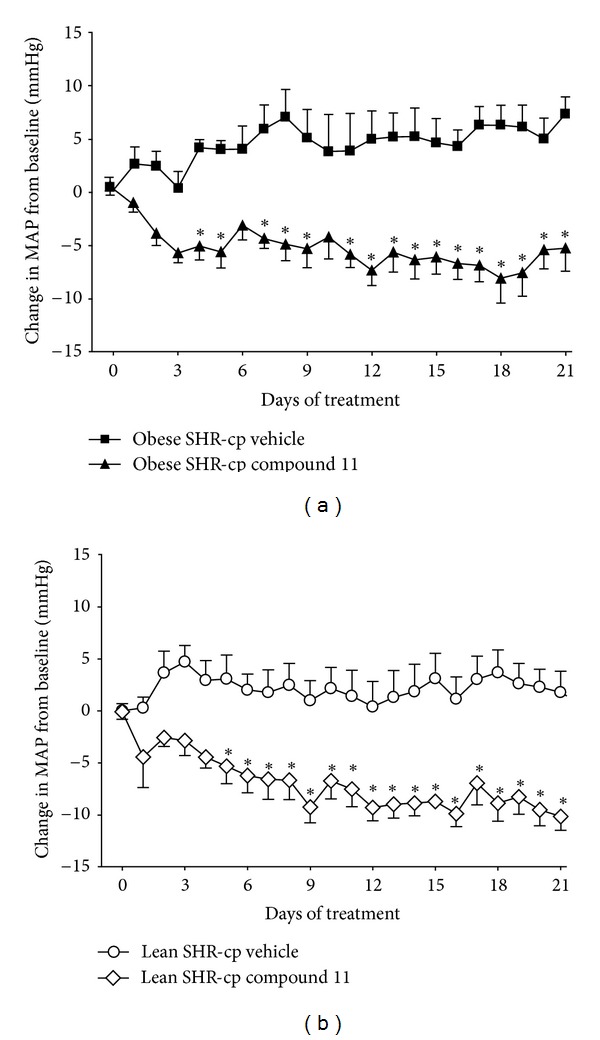
The change in mean arterial pressure (MAP) from baseline in obese (a) and lean (b) SHR-cp during three weeks of vehicle or compound 11 (10 mg/kg/d) administration. *P < 0.05 compared to vehicle.

The blood pressure and heart rate responses to chronic administration of compound 11 or vehicle in obese and lean SHR-cp are illustrated in Figures 1 and 2. At baseline, obese (126 ± 2 mmHg) and lean (144 ± 4 mmHg, *P* < 0.05 versus obese) SHR-cp have significantly higher mean arterial pressure (MAP) than age-matched WKY (105 ± 2 mmHg, data not shown). As shown in Figure 2, compound 11 administration decreased MAP similarly in obese and lean SHR-cp. Three weeks of compound 11 administration significantly decreased MAP in obese SHR-cp by an average of 5.7 ± 0.8 mmHg and in lean SHR-cp by an average of 7.3 ± 1.0 mmHg. In contrast, vehicle administration had no significant effect on MAP in obese (delta 4.5 ± 0.4 mmHg) and lean (delta 2.2 ± 0.2 mmHg) SHR-cp. The circadian rhythm of MAP was not altered by compound 11. Throughout the study, heart rate was significantly lower in obese compared to lean SHR-cp; compound 11 had no effect in either group.

The plasma lipid profiles of lean and obese SHR-cp before and after three weeks of vehicle or compound 11 administration are shown in Table 1. During baseline conditions, obese SHR-cp had significantly higher plasma cholesterol, low-density lipoprotein (LDL), high-density lipoprotein (HDL), and triglycerides compared to lean SHR-cp. Chronic administration of compound 11 had a significantly greater effect than vehicle treatment on the plasma lipid profile in lean and obese SHR-cp. In lean SHR-cp, compound 11 significantly decreased triglycerides by 30% and increased HDL by 25%, LDL by 138%, and total cholesterol by 49% from baseline. In obese SHR-cp, compound 11 significantly decreased plasma triglycerides by 59% and increased LDL by 274% and total cholesterol by 94% from baseline, with no change in HDL. Compound 11 significantly decreased triglycerides and increased LDL and subsequently total cholesterol more in obese than in lean SHR-cp. 

Table 2 summarizes the renal excretory responses to chronic administration of compound 11 or vehicle in obese and lean SHR-cp. During baseline conditions urine flow, electrolyte excretion, glucose excretion, and microalbumin excretion were significantly higher in obese compared to lean SHR-cp. However, there was no difference in creatinine clearance between groups, suggesting that glomerular filtration rates were similar in obese and lean SHR-cp. In lean SHR-cp, three weeks of compound 11 treatment had no significant effect on renal excretory function. In contrast, compound 11 significantly decreased urine flow by 44%, potassium excretion by 18%, and glucose excretion by 94% in obese SHR-cp. The effects of compound 11 on urine flow and glucose excretion were significantly greater than vehicle in obese SHR-cp. Chronic administration of compound 11 had no effect on sodium excretion, creatinine clearance, or microalbuminuria in obese SR-cp. 

**Figure 3:**
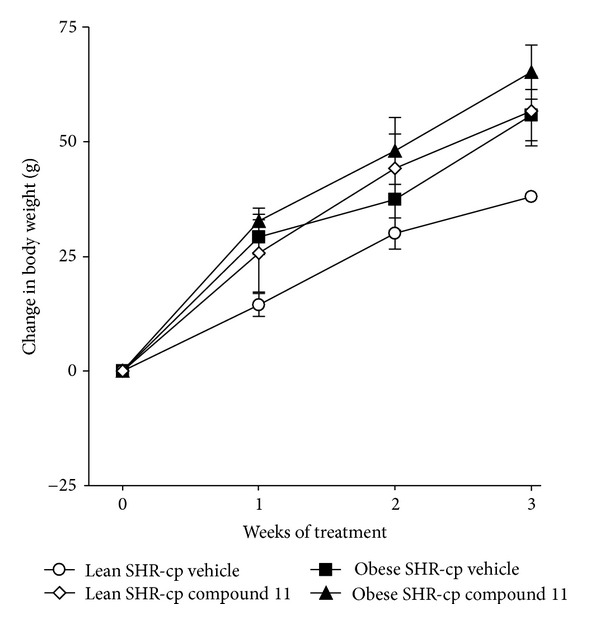
The change in body weight from baseline in obese and lean SHR-cp during three weeks of vehicle or compound 11 administration (10 mg/kg/d).

Figure 3 depicts the body weight response to compound 11 or vehicle administration in obese and lean SHR-cp. At baseline, obese SHR-cp had significantly higher body weight than lean SHR-cp (525 ± 12 g versus 383 ± 7 g). Three weeks of compound 11 administration at 10 mg/kg/d had no significant effect on body weight gain in obese or lean SHR-cp.

The renin-angiotensin-aldosterone system was assessed by measuring circulating renin and aldosterone concentrations before and after chronic vehicle or compound 11 administration. At baseline, the plasma aldosterone concentration was significantly higher in obese (273 ± 59 pg/mL) compared to lean (130 ± 26 pg/mL) SHR-cp. Compared to vehicle treatment, compound 11 had no significant effect on the plasma aldosterone concentrations in either lean (vehicle: 149 ± 28 pg/mL; compound 11: 186 ± 28 pg/mL) or obese (vehicle: 145 ± 26 pg/mL; compound 11: 184 ± 31 pg/mL) SHR-cp. However, plasma renin activity was significantly reduced in obese SHR-cp treated with compound 11 (vehicle: 10.7 ± 1.6 pg/mL; compound 11: 3.6 ± 0.4 pg/mL). 

### 3.2. Glucose Tolerance and Insulin Resistance

**Figure 4:**
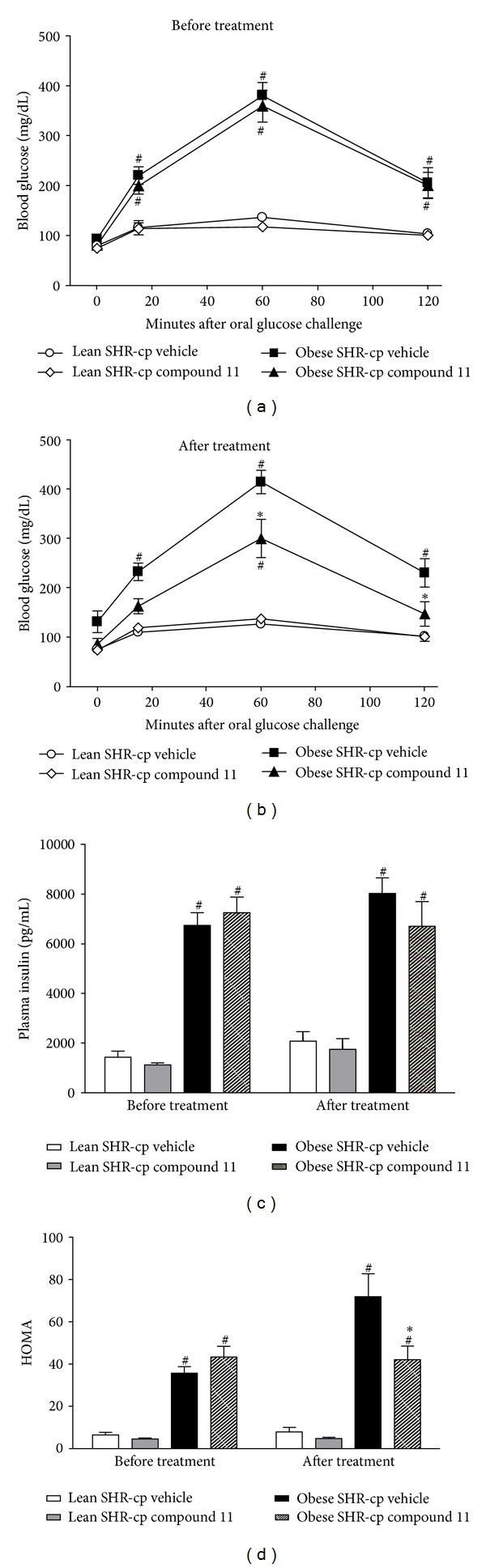
Indices of glucose tolerance and insulin resistance in obese and lean SHR-cp before and after 4 weeks of vehicle or compound 11 administration (10 mg/kg/d). (a) represents the blood glucose concentrations in all rats during oral glucose tolerance testing before vehicle or compound 11 administration. (b) represents the blood glucose concentrations in all rats during oral glucose tolerance testing after chronic vehicle or compound 11 administration. Oral glucose load was administered after fasting blood glucose was measured at *t* = 0 minutes. (c) is the plasma insulin concentration in all groups of rats before and after chronic vehicle or compound 11 treatment. (d) is the homeostasis model assessment index (HOMA) in all groups of rats before and after chronic vehicle or compound 11 treatment. *P < 0.05 compared to obese SHR-cp vehicle. ^#^ *P* < 0.05 compared to lean SHR-cp.

At baseline, obese SHR-cp had impaired glucose tolerance and insulin resistance compared to lean SHR-cp. The fasting blood glucose concentrations at baseline were similar among obese (vehicle: 93 ± 8 mg/dL; compound 11: 81 ± 8 mg/dL) and lean (vehicle: 79 ± 5 mg/dL; compound 11: 75 ± 4 mg/dL) SHR-cp. However, the fasting blood glucose response to glucose challenge was significantly impaired in obese compared to lean SHR-cp (see Figure 4(a)). The maximum blood glucose concentration that was achieved and the area under the oral glucose tolerance test curve (obese vehicle: 20151 ± 1722 mg/dL/min; obese compound 11: 19676 ± 1482 mg/dL/min; lean vehicle: 4399 ± 856 mg/dL/min; lean compound 11: 3893 ± 67 mg/dL/min) were significantly higher in obese than in lean SHR-cp. At baseline, plasma insulin concentrations (obese vehicle: 6746 ± 508 pg/mL; obese compound 11: 7257 ± 620 pg/mL; lean vehicle: 1419 ± 260 pg/mL; lean compound 11: 1112 ± 88 pg/mL) and the homeostasis model assessment-insulin resistance index (HOMA; obese vehicle: 36 ± 3; obese compound 11: 43 ± 5; lean vehicle: 6 ± 1; lean compound 11: 5 ± 0) were significantly elevated in obese compared to lean SHR-cp (Figures 4(c) and 4(d)). 

Four weeks of compound 11 administration significantly improved glucose tolerance and reduced insulin resistance in obese SHR-cp. The maximum blood glucose concentration in response to glucose challenge (Figure 4(b)), the area under the oral glucose tolerance test curve, and HOMA (Figure 4(d)) were significantly lower in obese SHR-cp chronically treated with compound 11 compared to vehicle. In contrast, compound 11 had no effect on glucose tolerance or HOMA in lean SHR-cp.

### 3.3. 11*β*-Hydroxysteroid Dehydrogenase Type 1 and Type 2 Activities

**Figure 5:**
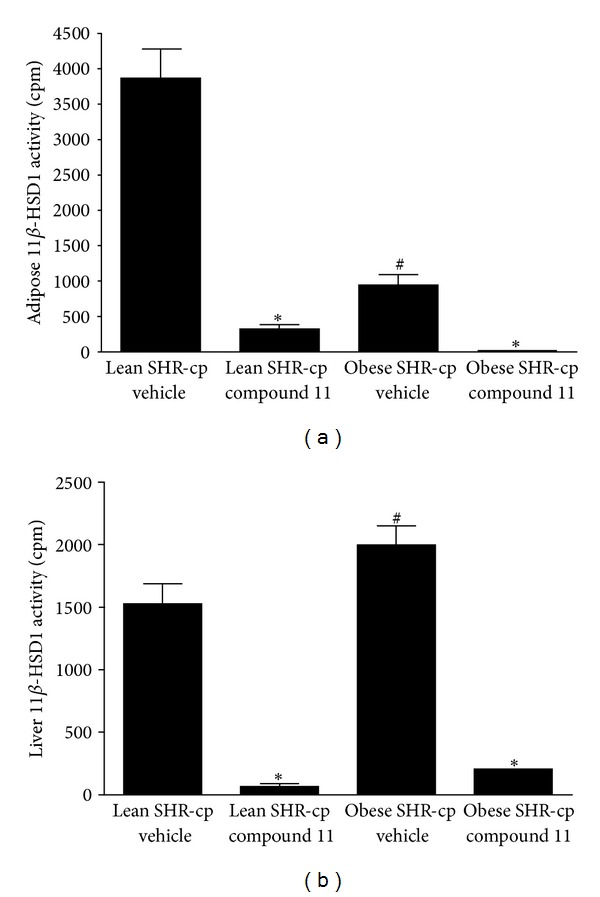
Bar graph depicts 11*β*-HSD1 activity in (a) adipose tissue and (b) liver of lean and obese SHR-cp after four weeks of vehicle or compound 11 (10 mg/kg/d) administration. *P < 0.05 compared to vehicle treatment of same genotype. ^#^ *P* < 0.05 compared to lean SHR-cp vehicle.

Figure 5 illustrates 11*β*-HSD1 cortisone reductase activities in adipose tissue and liver tissues of lean and obese SHR-cp after chronic treatment with vehicle or compound 11. After four weeks of treatment with vehicle, 11*β*-HSD1 activity in obese SHR-cp was significantly higher by 31% in liver and lower by 76% in adipose tissue compared to lean SHR-cp. However, obese SHR-cp had a much greater abundance of visceral fat than lean SHR-cp (authors' observations at tissue harvest), so total adipose 11*β*-HSD1 activity may have been elevated in obese compared to lean SHR-cp. In lean SHR-cp with compound 11 treatment, 11*β*-HSD1 activity was significantly lower by 96% in liver and by 92% in adipose tissue compared to vehicle. Similarly, in obese SHR-cp with compound 11 treatment, 11*β*-HSD1 activity was significantly lower by 90% in liver and by 97% in adipose tissue compared to vehicle. The cortisol dehydrogenase activity of 11*β*-HSD2 in kidney was similar between lean (4587 ± 98 cpm) and obese (4228 ± 113 cpm) SHR-cp with vehicle treatment, and compound 11 had no effect (lean: 4629 ± 459 cpm; obese: 4762 ± 148 cpm).

## 4. Discussion

 The major objective of this study was to determine the integrated cardiovascular, renal, and metabolic response to 11*β*-HSD1 inhibition in metabolic syndrome. Previous studies using either genetic models or pharmacological blockade uncovered a role for 11*β*-HSD1 in one or more of the risk factors in metabolic syndrome. However, no earlier study had investigated the comprehensive response, including blood pressure and renal function, to determine whether 11*β*-HSD1 is a common mechanism in the multiple risk factors. The results from this study show for the first time that pharmacological inhibition of 11*β*-HSD1 activity alone in adipose tissue and liver significantly decreases hypertension in a preclinical model of metabolic syndrome. Inhibition of 11*β*-HSD1 also reduces the glucose intolerance, insulin resistance, and elevated plasma triglycerides in metabolic syndrome. Together, these results expand upon previously published results and indicate that 11*β*-HSD1 is a common mechanism that contributes to the interrelated risk factors of metabolic syndrome.

Clinical studies previously have shown an association between glucocorticoids and hypertension in the presence or absence of metabolic syndrome. In humans with essential hypertension, the vasoconstrictor sensitivity to glucocorticoids is increased [33] and the ratio of excreted cortisol to cortisone metabolites is increased in some but not all cases [34–36]. Patients with the syndrome of apparent mineralocorticoid excess which is caused by a reduction in the peripheral metabolism of cortisol are hypertensive [18, 19]. Glucocorticoid sensitivity and salivary cortisol concentration are increased in hypertensive humans with insulin resistance and hyperglycemia, as well as in men with a predisposition to high blood pressure [37, 38]. These observations all suggest a primary role for glucocorticoids in the development of hypertension. 

 Not only there is a strong association between glucocorticoids and hypertension, but also clinical trials have shown a therapeutic benefit of reducing glucocorticoid activity on blood pressure, in some cases on a background of metabolic syndrome. Hypertension associated with Cushing's syndrome was reversed with the antiglucocorticoid agent RU486 [6]. Recently, Feig et al. reported that patients with type 2 diabetes mellitus and metabolic syndrome had a modest but significantly reduced blood pressure after twelve weeks of treatment with the 11*β*-HSD1 inhibitor MK-0916 [23]. Interestingly, in overweight and obese patients with hypertension, MK-0916 at the same dose had no significant effect on the primary endpoint of sitting blood pressure, but modestly improved other blood pressure endpoints [24]. In association with the small decrease in blood pressure, 11*β*-HSD1 inhibition also reduced elevated LDL and body weight in these clinical studies using small cohorts [23, 24].

Prior preclinical studies using the genetic manipulation of 11*β*-HSD1 suggested that the local (nonadrenal) generation of cortisol plays an important role in the regulation of blood pressure in metabolic syndrome (see [39] for review). Paterson et al. reported that liver-selective overexpression of 11*β*-HSD1 in mice caused a transgene dose-related increase in blood pressure [20]. Furthermore, mice with overexpression of 11*β*-HSD1 specifically in adipose tissue also exhibited high blood pressure [21]. In contrast to studies that increased the mRNA expression of 11*β*-HSD1 in selective tissues, Kotelevtsev et al. reported that the mean arterial pressure of 11*β*-HSD1^−/−^ mice is the same as in wild-type mice [22]. Other mechanisms may be compensating in the 11*β*-HSD1^−/−^ mice in order to maintain blood pressure. These preclinical studies suggested that increased 11*β*-HSD1 activity can markedly elevate blood pressure, but is not required for hypertension. We show for the first time that the pharmacological inhibition of 11*β*-HSD1 activity alone in liver and adipose tissue is sufficient to reduce blood pressure in an animal model of metabolic syndrome. 

Glucocorticoids have a wide range of activities within the cardiovascular, renal, and endocrine systems. Previous studies have shown that glucocorticoids can affect insulin resistance, gluconeogenesis, liposynthesis, accumulation of visceral fat, vascular reactivity, vascular remodeling, renal sodium reabsorption, and blood pressure [5–9, 40, 41]. However, the mechanisms whereby glucocorticoids can cause hypertension in humans remain unclear. Most evidence suggested that glucocorticoids increase blood pressure through the modulation of vascular structure and function, renal sodium reabsorption, and metabolic changes [5–9, 40, 41]. In this study, we examined whether pharmacological inhibition of 11*β*-HSD1 decreases blood pressure through a natriuretic mechanism. The results indicate that inhibition of 11*β*-HSD1 activity tended to decrease sodium and chloride excretion in obese SHR-cp, an effect opposite to a natriuretic agent. However, the small reduction in salt excretion could have been due to the significant decrease in urine flow, which was secondary to the reduced glucose excretion, since glucose is an osmotically active solute in renal tubules. 

An alternative explanation for the reduction in blood pressure may be found in the metabolic changes associated with inhibition of 11*β*-HSD1 activity. The present data clearly indicate that long-term inhibition of 11*β*-HSD1 significantly improves glucose tolerance and reduces insulin resistance, which may contribute to the lowering of blood pressure. Previous studies have demonstrated that reducing 11*β*-HSD1 activity decreased glucose intolerance and hyperinsulinemia in diet-induced obese mice [8, 42] and streptozotocin-induced diabetic mice [8]. However, blood pressure was not reported in those studies, thus rendering impossible any interpretation of the effects on insulin resistance (with or without glucose intolerance) on blood pressure. Whether hyperinsulinemia per se causes chronic elevations in blood pressure is still controversial and may be dependent upon the species studied [43].

Our data suggests that inhibition of 11*β*-HSD1 decreases blood pressure in hypertensive SHR-cp independent of metabolic changes. Inhibition of 11*β*-HSD1 in adipose tissue and liver of obese SHR-cp decreases glucose tolerance, insulin resistance, hypertriglyceridemia, and hypertension. Yet in lean SHR-cp, inhibition of 11*β*-HSD1 similarly decreases hypertension in the absence of changes in glucose tolerance or insulin resistance. With the caveat that the metabolic response to 11*β*-HSD1 inhibition may simply not be manifested in lean SHR-cp because of their normal metabolic state, our results suggest that 11*β*-HSD1 is an independent mediator of hypertension in SHR-cp. Regardless, 11*β*-HSD1 is a common mechanism in multiple risk factors in metabolic syndrome. 

Finally, the mechanism for the reduction of high blood pressure likely includes modulation of the renin-angiotensin system. Previous investigators showed that glucocorticoids increased hepatic synthesis of angiotensinogen [44] and angiotensin II receptor subtype 1 in peripheral tissues [45]. Indeed, mice overexpressing 11*β*-HSD1 activity in adipose tissue had increased plasma angiotensinogen, angiotensin II, and aldosterone concentrations and are hypertensive. The elevated blood pressure was abrogated by blockade of the angiotensin II type 1 receptor [22]. Independent studies of the corpulent SHR showed that blockade of angiotensin II type 1 receptor [46] or mineralocorticoid receptor [25, 47], inhibition of angiotensin converting enzyme [48], or antioxidant therapy [49, 50] all significantly decreased blood pressure indicating that angiotensin II and its downstream effects mediated the hypertension. The decreased plasma renin activity in our study suggests that the antihypertensive response to 11*β*-HSD1 inhibition is likely due, at least in part, to a reduction in angiotensin II actions. 

## 5. Conclusion

The present study shows that inhibition of 11*β*-HSD1 activity decreases hypertension, insulin resistance, glucose intolerance, and hypertriglyceridemia in obese SHR-cp. These are prominent features of the metabolic syndrome, and 11*β*-HSD1 appears to be a common regulatory mechanism among them. Longitudinal clinical studies have confirmed that metabolic syndrome is a risk factor for subsequent development of cardiovascular disease and mortality [51]. The prevalence of metabolic syndrome has increased over decades among adults in the United States [4]. Although adults with hypertension are more likely to be insulin resistant [52, 53] and hypertension tends to cluster with other metabolic risk factors [54], there are currently no guidelines for treating hypertension specifically in individuals with metabolic syndrome. The present study provides preclinical support for the pharmacological inhibition of 11*β*-HSD1 for the treatment of hypertension and other interrelated risk factors in metabolic syndrome.

## Figures and Tables

**Table 1:** Plasma lipid profile in lean and obese SHR-corpulent rats before and after three weeks of treatment with vehicle or compound 11.

	Period	Lean SHR-cp vehicle	Lean SHR-cp compound 11	Obese SHR-cp vehicle	Obese SHR-cp compound 11
Total cholesterol (mg/dL)	Baseline	64 ± 2	62 ± 1	122 ± 7^#^	121 ± 8^#^
	Week 3	74 ± 3	93 ± 3*	154 ± 6^#^	233 ± 14^#∗^
LDL cholesterol (mg/dL)	Baseline	8 ± 1	9 ± 1	14 ± 1^#^	15 ± 2^#^
	Week 3	16 ± 1	20 ± 1*	34 ± 2^#^	55 ± 5^#∗^
HDL cholesterol (mg/dL)	Baseline	21 ± 0	21 ± 1	35 ± 2^#^	34 ± 1^#^
	Week 3	23 ± 1	25 ± 1*	42 ± 2^#^	45 ± 2^#^
Triglycerides (mg/dL)	Baseline	60 ± 4	60 ± 3	516 ± 79^#^	662 ± 139^#^
	Week 3	62 ± 8	42 ± 3*	654 ± 91^#^	253 ± 31^#∗^
NEFA (mmol/L)	Baseline	1.6 ± 0.1	1.7 ± 0.1	1.4 ± 0.1	1.4 ± 0.1
	Week 3	1.0 ± 0.2	0.7 ± 0.1	1.1 ± 0.1	1.0 ± 0.1

Data are expressed as mean ± SEM. LDL: low-density lipoprotein; HDL: high-density lipoprotein; NEFA: nonesterified fatty acid. *P < 0.05 versus vehicle within genotype; ^#^ *P* < 0.05 versus lean SHR-cp.

**Table 2:** Renal excretory function in lean and obese SHR-corpulent rats before and after three weeks of treatment with vehicle or compound 11.

	Period	Lean SHR-cp vehicle	Lean SHR-cp compound 11	Obese SHR-cp vehicle	Obese SHR-cp compound 11
Urine flow (mL/day)	Baseline	18 ± 1	21 ± 2	57 ± 4^#^	56 ± 6^#^
	Week 3	24 ± 2	26 ± 3	46 ± 4^#^	30 ± 4*
Sodium excretion (mmol/day)	Baseline	2.2 ± 0.1	2.3 ± 0.2	3.5 ± 0.2^#^	3.1 ± 0.2^#^
	Week 3	2.4 ± 0.1	2.0 ± 0.2	3.2 ± 0.3^#^	2.7 ± 0.3^#^
Potassium excretion (mmol/day)	Baseline	5.0 ± 0.3	5.1 ± 0.3	8.4 ± 0.3^#^	8.3 ± 0.3^#^
	Week 3	5.6 ± 0.2	4.8 ± 0.5	8.0 ± 0.6^#^	6.7 ± 0.4^#∗^
Chloride excretion (mmol/day)	Baseline	3.7 ± 0.2	3.7 ± 0.3	5.8 ± 0.2^#^	5.3 ± 0.3^#^
	Week 3	3.7 ± 0.1	3.1 ± 0.3	5.3 ± 0.5^#^	4.6 ± 0.2^#^
Glucose excretion (mg/day)	Baseline	7 ± 2	9 ± 1	3568 ± 391^#^	3638 ± 538^#^
	Week 3	6 ± 2	4 ± 1	1148 ± 220^#^	220 ± 156^#∗^
Creatinine clearance (L/day)	Baseline	6.00 ± 0.56	5.86 ± 0.20	5.47 ± 0.35	5.14 ± 0.20
	Week 3	6.28 ± 0.60	5.08 ± 0.77	5.95 ± 1.36	5.00 ± 0.23
Microalbumin excretion (mg/day)	Baseline	2 ± 0	1 ± 0	49 ± 15^#^	50 ± 22^#^
	Week 3	5 ± 1	10 ± 8	85 ± 28^#^	77 ± 28^#^

Data are expressed as mean ± SEM. *P < 0.05 versus obese SHR-cp vehicle; ^#^ *P* < 0.05 versus lean SHR-cp.
